# Hepatitis C Virus-Induced FUT8 Causes 5-FU Drug Resistance in Human Hepatoma Huh7.5.1 Cells

**DOI:** 10.3390/v11040378

**Published:** 2019-04-24

**Authors:** Shu Li, Xiao-Yu Liu, Qiu Pan, Jian Wu, Zhi-Hao Liu, Yong Wang, Min Liu, Xiao-Lian Zhang

**Affiliations:** State Key Laboratory of Virology, Department of Immunology, Hubei Province Key Laboratory of Allergy and Immunology and Medical Research Institute, Wuhan University School of Basic Medical Sciences, Wuhan 430071, China; 2015103010009@whu.edu.cn (S.L.); xiaoyu__liu@yeah.net (X.-Y.L.); panqiu15927308377@gmail.comm (Q.P.); JianWu@whu.edu.cn (J.W.); lzhihao2016@126.com (Z.-H.L.); yongwangmmm@gmail.com (Y.W.)

**Keywords:** hepatitis C virus (HCV), α-1,6 fucosyltransferase (FUT8), 5-fluorouracil (5-FU), drug-resistance

## Abstract

Hepatitis C virus (HCV) is a major cause of human chronic liver disease and hepatocellular carcinoma. Our recent studies showed that α1,6-fucosyltransferase (FUT8), a key glycosyltransferase, was the most up-regulated glycosyltransferase after the HCV infection of human hepatocellular carcinoma Huh7.5.1 cells. Here, we further studied the effects and possible mechanism of FUT8 on the proliferation of HCV and chemotherapy-resistance of HCV-infected Huh7.5.1 cells. The effects of FUT8 on the proliferation and drug resistance of HCV-infected Huh7.5.1 cells were analyzed by flow cytometry analysis (FCM), quantitative real-time polymerase chain reaction (qRT-PCR), Western blot analysis and lactate dehydrogenase (LDH) release assay. Results: We found that FUT8 not only promoted Huh7.5.1 proliferation by activating PI3K-AKT-NF-κB signaling, but also stimulated the expression of the drug-resistant proteins P-glycoprotein (P-gp) and multidrug resistance related protein 1 (MRP1) and enhanced the 5-fluorouracil (5-FU) chemo-resistance of Huh7.5.1 cells. Silencing of FUT8 reduced the cell proliferation and increased the 5-FU sensitivity of HCV-infected Huh7.5.1 cells. Inhibition of P-gp and MRP1 increased the 5-FU drug sensitivity in HCV infected Huh7.5.1 cells. HCV-induced FUT8 promotes proliferation and 5-FU resistance of Huh7.5.1 cells. FUT8 may serve as a therapeutic target to reverse chemotherapy resistance in HCV-infected Huh7.5.1 cells.

## 1. Introduction

Hepatitis C virus (HCV) infection has become a major public health problem and currently affects more than 180 million people worldwide [1]. HCV typically causes a chronic infection and is a major cause of cirrhosis, end-stage liver disease, and hepatocellular carcinoma (HCC) [2].

Altered *N*-glycosylation and glycosyltransferase expression in tumor cells is implicated in cancer progression, metastasis and therapeutics [3]. Glycosylation is catalyzed by various glycosyltransferase enzymes, which glycosylate various complex carbohydrates such as glycoproteins, glycosphingolipids, and proteoglycans. Aberrant glycosylation and increased fucosylation are recognized as an indicator of tumors [4,5]. The fucosyltransferase (FUT) family is a group of fucosylation synthases. By catalyzing the transfer of the fucose (Fuc) residue from the donor substrate, GDP-Fuc, to the oligosaccharide acceptor in a1,2-(FUT1 and FUT2), a1,3/4-(FUT3, FUT4, FUT5, FUT6, FUT7, FUT9, FUT10 and FUT11) and a1,6-linkages (FUT8), FUTs promote the synthesis of fucosylated oligosaccharides on glycoconjugates. Fucosylated oligosaccharides have been implicated in multiple types of cell–cell interactions in differentiation, development and malignancy. Forced FUT1 and FUT2 expression in human ovarian carcinoma-derived RMG-I cells promoted cell proliferation and resistance against anticancer drugs, such as 5-fluorouracil (5-FU) and carboplatin [6]. Fucosyltransferase 4 (FUT4)-catalyzed fucosylated *N*-glycans were dramatically increased in multidrug-resistant breast cancer cells [7]. The altered levels of FUT4, FUT6 and FUT8 were responsible for drug-resistant phenotypes of BEL7402 and BEL/FU cells both in vitro and in vivo [8]. FUT8 has been reported as a driver of melanoma metastasis and silencing of FUT8-suppressed invasion and tumor dissemination [9].

To date, 5-FU is one of important chemotherapeutic drugs used for the treatment of cancer, including HCC [10]. Multiple mechanisms have been proposed to explain 5-FU resistance [11,12]. The uptake of 5-FU by cancer cells is mainly carried out through concentrative nucleoside transporter 1, whose expression is downregulated in HCC [13]. Overexpression of thymidylate synthase has been found to be associated with 5-FU resistance in colorectal cancer [12]. Glucose transporter type 1 (Glut1) expression was reported to be upregulated in 5-FU resistant cancer cells, and exogenous overexpression of Glut1 facilitated colorectal cancer cells obtaining resistance to 5-FU [14].

Recently, the aberrant glycosylation of tumor cells has been implicated in the resistance to chemotherapy of malignant cells [15]. However, the effects of FUT8 on the drug resistance of HCC, especially HCC caused by HCV infection, have not yet been clearly defined.

Here, we studied the effects and possible mechanism of FUT8 on the proliferation of HCV and the chemo-resistance of Huh7.5.1 cells. Our results provide evidence that FUT8 causes cell proliferation and 5-FU drug resistance in human hepatoma Huh7.5.1 cells after HCV infection.

## 2. Materials and Methods

### 2.1. Cell Culture, Viral Infection and Reagents

Huh7.5.1 cells were cultured in complete Dulbecco’s modified Eagle’s medium (DMEM) with 10% fetal bovine serum (FBS) (HycloneLaboratories, Logan, UT, USA). The JFH1 (Japanese fulminant hepatitis 1, genotype 2a) HCV cell culture (HCVcc) infection was performed according to our previous publication [16,17,18]. In brief, Huh7.5.1 cells in 24-well plates were infected with HCVcc (multiplicity of infection of ~1; 1 × 10^7^ copies/mL) at 37 °C for 4 h. The supernatants were discarded, and the infected cells were washed twice with phosphate buffered saline (PBS) and incubated in DMEM containing 10% FBS for each experiment. P-glycoprotein (P-gp) inhibitor NSC23925 and multidrug resistance-associated protein-1 (MRP1) inhibitor MK571 were purchased from MedChemExpress (MCE) (Monmouth Junction, NJ, USA) and dissolved in dimethyl sulfoxide (DMSO). Five-fluorouracil (5-FU), methotrexate (MTX), cisplatin (DDP, CDDP) and adriamycin (ADR) were purchased from Target Mol (Boston, MA, USA). Monoclonal antibodies anti-P-gp (E1Y7B) and anti-MRP1 (D7O8N), anti-phospho-Akt (Ser473), anti-β-actin and polyclonal anti-PI3 kinase p110 were purchased from Cell Signaling Technology (Danvers, CA, USA). Anti- reduced glyceraldehyde-phosphate dehydrogenase (GAPDH) antibody was from Proteintech Group Inc (Rosemont, IL, USA). Polyclonal anti-FUT8 and monoclonal anti-NS3 were from Abcam (Cambridge, UK). Monoclonal anti-NF-κB p-p65 (93H1) was from Santa Cruz Biotechnology (Dallas, TX, USA).

### 2.2. Small Interfering RNA (SiRNA) Transfection

Transfection was performed in Huh7.5.1 cells in a 6-well plate at 30%–50% confluency. Huh7.5.1 cells were maintained in 1 ml of complete medium with 10^7^ copies of HCVcc per well overnight and then were transfected with FUT8-specific siRNA (sense: 5’-UUCCUGGCGUUGGAUUAUGCUCAUU-3’; antisense: 5’-AAUGAGCAUAAUCCAACGCCAGGAA-3’) or scrambled siRNA as siRNA control (sense: 5’-UUCUCCGAACGUGUCACGUTT-3’; antisense: 5’-ACGUGACACGUUCGGAGAATT-3’). FUT8 siRNA was mixed with INTERFERin^®^ in vitro siRNA transfection (Invitrogen, Carlsbad, CA, USA). Transfected cells were cultured and incubated at 37 °C for 6 h, followed by incubation with complete medium for an additional 48 h. Cells were then harvested for further study. All experiments were performed at least three separate times.

### 2.3. Plasmid Construction and Transfection

The human full-length FUT8 cDNA (GenBank accession no. NM_004480.4) was subcloned in-frame into pcDNA3.1(−)Myc-His(−)A, at the sites *Eco*RI and *Bam*HI. The specific primers were as follows: primer F (5’-CGGAATTCCGATGACGGATCTATACTACCTCAGTC-3’) and primer R (5’-CGGGATCCTTTCTCAGCCTCAGGATATGT-3’). The pcDNA3.1-FUT8 plasmid containing the FUT8 coding sequence was then verified by DNA sequencing. For plasmid transfection, Huh7.5.1 cells were seeded into 6-well plates 24 h before transfection with each plasmid (1 μg/well) using Lipofectamine 2000 Reagent (Invitrogen, USA), according to the manufacturer’s instructions. The pcDNA3.1 was used as an empty vector control. After 48 h of transfection, the cells were collected for further studies. The cell transfection efficiency was 70%, and the survival rate was 90%.

### 2.4. Quantitative Real-Time Polymerase Chain Reaction (qRT-PCR)

QRT-PCR was used to analyze mRNA expression. Huh7.5.1 cells or HCVcc-infected Huh7.5.1 (2 × 10^7^ each) were pre-transfected with or without FUT8-siRNA. For the FUT8 overexpression group, Huh7.5.1 cells were transfected with the pcDNA3.1-FUT8 plasmid or the pcDNA3.1 vector. After 48 h, total RNA was isolated with Trizol reagent (Invitrogen, USA) and cDNA was synthesized using the ReverTra Ace–First Strand cDNA Synthesis kit (Toyobo, Japan) according to the manufacturer’s protocol. RT-qPCR was carried out on a StepOnePlus Real-Time PCR System (Applied Biosystems, Foster City, CA, USA) using SYBR^®^ Green Real-Time PCR Master Mix (Toyobo, Japan). The relative mRNA expression levels of each gene were normalized based on GAPDH. Relative fold differences were determined using the method of delta–delta CT, calculated as 2^−(Ct^_Target gene_^−Ct^_GAPDH_^)^. All experiments were performed at least three separate times.

### 2.5. Lactate Dehydrogenase Release Assay

The lactate dehydrogenase release (LDH) assay was used to evaluate the IC50 value (drug concentration that inhibits cell growth by 50%) of the drug. The detection of LDH released from the cells was performed according to the manufacturer’s instructions (CytoTox 96^®^Non-Radioactive Cytotoxicity Assay kit, G1780, Promega, USA). Briefly, HCVcc-infected Huh7.5.1 or Huh7.5.1 cells (1 × 10^5^ each) were added into each six-well plate. After 48 h, each group of cells were seeded in 96-well plates at 5000 cells/well. Then, the cells were treated with different concentrations of anti-tumor drugs as indicated. Forty-eight hours later, the cells were lysed with lysis solution. After centrifuging, the supernatant (50 µL) was transferred to an enzymatic assay plate, followed by the addition of 50 µL/well of reconstituted substrate mix. After incubation at room temperature for 30 min, stop solution was added to each well. Absorbance was recorded at 490 nm. The percentage of cytotoxicity was calculated according to the following formula: % cytotoxicity = (experimental LDH release (OD_490_)–spontaneous LDH release (OD_490_))/(maximum LDH release (OD_490_)–spontaneous LDH release (OD_490_)) × 100.

In order to detect the effect of FUT8 on the anti-tumor drug sensitivity, HCVcc-infected Huh7.5.1 or Huh7.5.1 cells were added into six-well plate (1 × 10^5^ cells/well) and tranfected with FUT8 siRNA or control siRNA. To detect the effect of MRP1 and P-gp inhibitors on the anti-tumor drug sensitivity, HCVcc-infected Huh7.5.1 or Huh7.5.1 cells in 6-well plates were treated with MRP1 inhibitor MK571 or P-gp inhibitor NSC23925. 70 µM MK571, and 1 µM NSC23925 were used as work concentrations according to previous reports [19]. After 12 h, each group of cells were seeded in 96-well plates and afterwards exposed to different concentrations of anti-tumor drugs. After 12 h, the cells were lysed and examined with LDH kit as described above. Each sample consisted of four replicate wells, and the result was expressed as the mean value ± standard deviation (SD). All experiments were performed at least three separate times. All data were analyzed for statistical significance.

### 2.6. Cell Proliferation Assay

Cell proliferation was measured via cell viability using a Cell Counting Kit-8 (CCK8, Dojindo, Japan) and conducted as previously described [20]. Huh7.5.1 cells were pretreated with PI3K inhibitors (LY294002, Sigma, USA) or DMSO reagent control. After siRNA and plasmid transfection for 24 h, the cells were infected with HCVcc for different time periods (0 h, 24 h, and 48 h). A total of 10 µL CCK8 reagents were added and kept in the incubator at 37 °C for 4 h. Finally, the absorbance was determined at 450 nm by a Microplate Reader (iMarkTM, Bio-Rad, Alfred Nobel Drive Hercules, CA, USA). There are 5 replicate wells for each sample. Three independent experiments were performed for each analysis.

### 2.7. Western Blot Analysis

HCVcc-infected cells or Huh7.5.1 cells (2 × 10^7^ each) were transfected with pcDNA3.1-FUT8, FUT8-siRNA or empty vector. After 12 h, the cells were lysed with tissue protein extraction reagent (T-PER) (Thermo Scientific; San Jose, CA, USA) according to the manufacturer’s instructions. Protein concentration was measured by the BCA kit (Beyotime, Shanghai, China). Whole cell lysates were electrophoresed on a 10% SDS-PAGE gel and blotted onto a polyvinylidene difluoride membrane. After blocking with 5% BSA in TBS (20 mMTris, 150 mMNaCl, pH 7.4) containing 0.05% Tween 20 (TBST), the membrane was incubated with the antibody of interest (Santa Cruz Biotechnology, Santa Cruz, CA, USA, 1:200 dilution; Cell Signaling Technology, 1:1000 dilution; Abcam, Cambridge, UK, 1:1000 dilution) and then with peroxidase-conjugated anti-rabbit IgG (Santa Cruz Biotechnology, Santa Cruz, CA, USA, 1:3000 dilution). β-actin (Santa Cruz Biotechnology, 1:2000 dilution) was used as a control. All band intensities were evaluated using an ECL Western blotting kit (Millipore, Belmopán, Belize) and were normalized to those of β-actin. Three independent experiments were performed for each analysis.

To elucidate the signaling pathway involved in the FUT8-mediated drug resistance in Huh7.5.1 cells, Huh7.5.1 cells were treated with specific inhibitors of PI3K (LY294002), NK-κB (Bay11-7082) and p-MEK (PD98059) for 2 h. The cells were then either infected with HCV or transfected with pcDNA3.1-FUT8 or the pcDNA3.1 vector.

To verify the effect of MRP1 inhibitor MK571 and P-gp inhibitor NSC23925, Huh7.5.1 cells were treated with MK571 (70 µM) and NSC23925 (1 µM), respectively. After 12 h, the cells were harvested and cell lysates were analyzed by SDS-PAGE and Western blot using anti-P-gp and anti-MRP1, respectively. GAPDH was used as an internal control.

### 2.8. Flow Cytometry Analysis

HCVcc-infected cells or Huh7.5.1 cells (2 × 10^7^ each) were transfected with pcDNA3.1-FUT8, FUT8-siRNA or empty vector. After 48 h, the cells were harvested and washed with PBS buffer and then were fixed with 200 μL of fixation buffer and incubated at room temperature in the dark for 20 min. Cells were washed with BioLegend’s FOXP3 Perm buffer and then were placed in sterile conical tubes in aliquots of 5 × 10^5^ cells in 100 μL BioLegend’s FOXP3 Perm buffer each. For Ki67 staining, the cells were stained with the FITC-conjugated Ki67 antibody and incubated at room temperature in the dark for 30 min. The cells were then washed with BioLegend Cell Staining buffer and resuspended in 0.5 mL cell staining buffer for flow cytometric analysis. For the detection of P-gp and MRP1, the cells were stained with anti-MRP1, anti-P-gp rabbit mAbs or rabbit IgG (isotype) (Cell Signaling Technology) followed by fluorescein isothiocyanate (FITC)-conjugated goat IgG anti-rabbit IgG. Three independent experiments were performed for each analysis.

### 2.9. Statistical Analysis

All data are presented as the mean ± standard deviation (SD) and analyzed by GraphPad Prism V.5.00 software (GraphPad Software, San Diego, CA, USA). Differences between groups were tested by one-way ANOVA followed by the Newman–Keuls post hoc test. Two-sided *p*-values under 0.05 were considered statistically significant (*, *p* < 0.05; **, *p* < 0.01; ***, *p* < 0.001). NS represents no significance.

## 3. Results

### 3.1. HCV Infection Induces FUT8 Expression in Huh7.5.1 Cells

**Figure 1:**
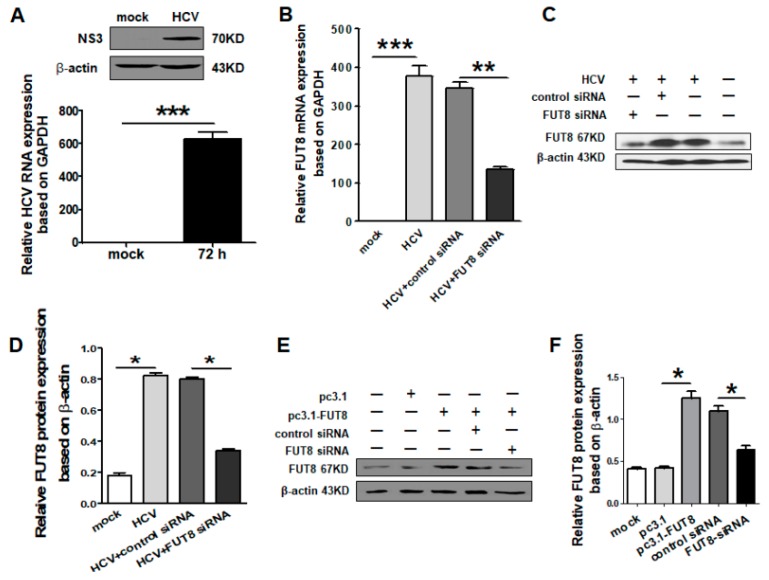
FUT8-specific siRNA and recombinant expression vector were designed and verified. (**A**) qRT-PCR and Western blotting analysis were used to detect the relative hepatitis C virus (HCV) RNA expression and non-structural protein 3 (NS3) protein level 72 h post HCV infection in Huh7.5.1 cells. Mock: only Huh7.5.1 cells. The specificity of the FUT8 siRNA was confirmed by RT-PCR (**B**) and Western blot (**C**). Statistical analyses of (**C**) are listed in (**D**). (**E**) Overexpression of FUT8 was confirmed by Western blot after transfection with FUT8 plasmid (pcDNA3.1-FUT8, named pc3.1-FU8). Statistical analyses of (**E**) are also listed in (**F**).

Currently, the most commonly used infectious HCV culture system is based on JFH1, which undergoes efficient replication in human Huh-7 cells and other cell lines [21]. We analyzed HCV JFH1 RNA and NS3 protein expression levels in Huh7.5.1 cells by qRT-PCR and Western blot (Figure 1A). A significant increase in FUT8 mRNA and protein expression were observed in HCVcc-infected Huh7.5.1 cells (Figure 1A). In order to elucidate the direct implication of FUT8 on the proliferation and chemo-resistance of Huh7.5.1 cells, we designed and examined the effects of FUT8-specific siRNA. The mRNA expression of FUT8 was significantly decreased in the FUT8 siRNA-treated group compared with the control siRNA-treated group in the HCV-infected Huh7.5.1 cells (Figure 1B). At the same time, the protein level of FUT8 was significantly reduced in the FUT8-siRNA-treated group compared with the control siRNA-treated group (*, *p* < 0.05; Figure 1C,D). After transfection with the FUT8 expression vector (named pc3.1-FUT8), the protein level of FUT8 was much higher than after transfection with the empty vector control (Figure 1E,F). Transfection of FUT8-specific siRNA caused decreased expression of FUT8 (Figure 1E,F).

### 3.2. Both HCV Infection and Overexpression of FUT8 Enhanced Proliferation of Huh7.5.1 Cells

**Figure 2:**
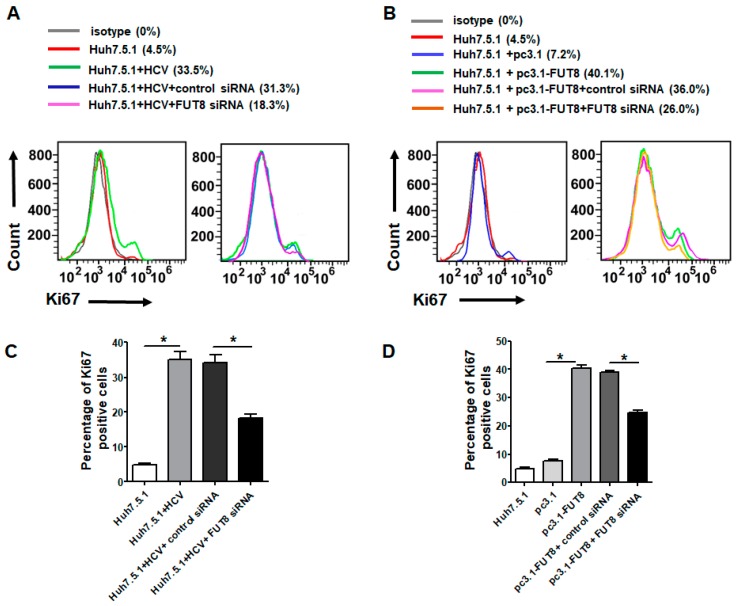
HCV infection and overexpression of FUT8 enhanced proliferation of Huh7.5.1 Cells. The expression levels of Ki67 were assessed by flow cytometry analysis (FCM). Huh7.5.1 cell proliferation can be observed after HCV infection for 72 h (**A**) or FUT8 overexpression (**B**), which could be inhibited by knockdown of FUT8. Statistical analyses of A and B are also listed as (**C**,**D**).

The Ki67 protein is a cellular marker for proliferation. To investigate whether FUT8 plays an important role in HCVcc stimulation, we analyzed the cellular Ki67 expression of Huh7.5.1 cells after HCV infection. Significantly higher levels of Ki67 were observed in HCVcc-infected Huh7.5.1 cells compared with control Huh7.5.1 cells by FCM analysis (HCVcc-infected Huh7.5.1 *vs*. Huh7.5.1, 33.5% *vs*. 4.5%) (Figure 2A). However, Ki67 expression was decreased by FUT8-siRNA knockdown (33.5% decreased to 18.3%) but was not affected by control siRNA (33.5% to 31.3%) (Figure 2A). Similarly, much higher levels of Ki67 were observed in the pc3.1-FUT8-transfected Huh7.5.1 cells compared with empty vector pc3.1-treated Huh7.5.1 cells by FCM analysis (pc3.1-FUT8-Huh7.5.1 *vs*. pc3.1-Huh7.5.1, 40.1% *vs*. 7.2%) (Figure 2B), and the increased Ki67 was decreased by subsequent silencing of FUT8 with FUT8-siRNA (40.1% to 26.0%), but not by control siRNA (40.1% to 36%) (Figure 2B). These data suggest that FUT8 is critical for HCV-induced cell proliferation (Figure 2A,C). Both HCV infection and overexpression of FUT8 stimulated Huh7.5.1 cell proliferation, and these effects can be inhibited by subsequent silencing of FUT8 (Figure 2B,C).

### 3.3. Silencing the FUT8 Gene Increases the 5-FU Drug Sensitivity of HCV-Infected and FUT8-Overexpressing Huh7.5.1 Cells

**Figure 3:**
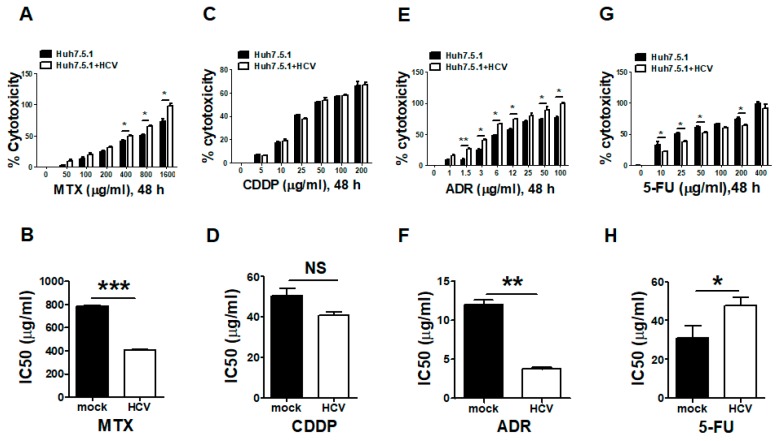
HCV infection caused 5-FU drug resistance. The effects of HCV infection on the chemosensitivity in Huh7.5.1 cells were evaluated using the lactate dehydrogenase (LDH) assay. The cytotoxicity and IC50 of MTX (**A**,**B**), CDDP (**C**,**D**), ADR (**E**,**F**) and 5-FU (**G**,**H**) in HCVcc-infected Huh7.5.1 cells was calculated using the LDH release assay.

Tumor chemotherapy drugs extensively tested in clinical trials, such as 5-FU, MTX, CDDP and ADR, can inhibit the proliferation of HCC. Since FUT8 was most upregulated by HCV infection in Huh7.5.1 cells, we examined the effects of FUT8 on the chemotherapy-resistance of Huh7.5.1 cells after HCV infection. The inhibitory effects of the drugs on the growth of HCVcc-infected Huh7.5.1 cells were evaluated using the LDH assay. We found that the cytotoxicity of MTX and ADR (Figure 3A,E) was increased and the IC50 values of MTX (Figure 3B, ***, *p* < 0.001) and ADR (Figure 3F, **, *p* < 0.01) were significantly decreased after HCV infection. There was no significant difference for CDDP (Figure 3C,D). However, the IC50 of 5-FU (Figure 3G–H) was remarkably increased in the HCVcc-infected Huh7.5.1 cells compared with the Huh7.5.1 cells, suggesting that HCV infection can induce 5-FU resistance of Huh7.5.1 cells. Consequently, 5-FU was selected as the target in the following experiment.

**Figure 4:**
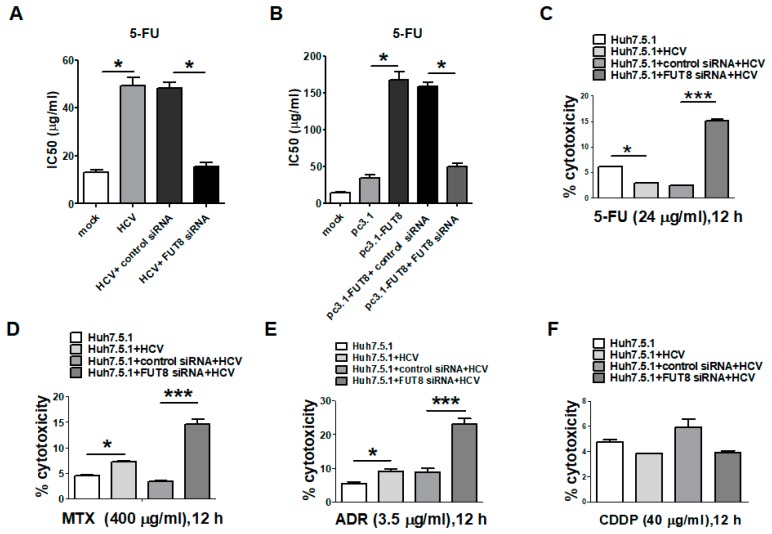
Silencing of FUT8 enhanced the chemosensitivity of Huh7.5.1 Cells. The effect of knockdown (**A**) or overexpression of FUT8 (**B**) on the chemosensitivity of 5-FU was also determined using the LDH assay. (**C**–**F**) The LDH release assay was used to analyze the cytotoxicity of the 5-FU/MTX/ADR/CDDP in HCVcc-infected Huh7.5.1 cells by knockdown of FUT8.

As shown in Figure 4A, the increase in the IC50 of 5-FU caused by HCV infection was inhibited by FUT8 knockdown (FUT8 siRNA *vs*. control siRNA, *, *p* < 0.05). We also observed that overexpression of FUT8 increased the drug resistance of Huh7.5.1 cells to 5-FU (pc3.1-FUT8 group *vs.* pc3.1 group, *, *p* < 0.05), which could be inhibited by FUT8 knockdown (FUT8 siRNA group *vs.* control siRNA pc3.1 group, *, *p* < 0.05; Figure 4B). The result of LDH assays also showed that HCV infection could decrease the cytotoxicity of 5-FU, which could be rescued by FUT8 knockdown (Figure 4C, FUT8 siRNA vs. control siRNA, ***, *p* < 0.001). The above data suggested that both HCV infection and FUT8 overexpression in Huh7.5.1 cells led to 5-FU drug resistance, while silencing of FUT8 gene enhanced the 5-FU drug sensitivity of HCV infection and FUT8 overexpression of Huh7.5.1 cells. However, HCV infection increased sensitivity to MTX and ADR (Figure 4D,E), and FUT8 knockdown further increased sensitivity to MTX and ADR in HCVcc-Huh7.5.1 cells (Figure 4D,E, FUT8 siRNA vs. control siRNA, ***, *p* < 0.001). No effect—or a limited effect—on the cytotoxicity and sensitivity to CDDP was observed by knockdown of FUT8 (Figure 4F). These data suggested that HCV infection and FUT8 had different effects on these anti-tumor drugs. HCV infection and FUT8 overexpression only led to 5-FU drug resistance, while knockdown of FUT8 increased the drugs’ sensitivity to 5-FU, MTX and ADR.

### 3.4. Upregulation of the Drug Resistance Genes P-gp and MRP1 after HCV Infection and FUT8 Overexpression

**Figure 5:**
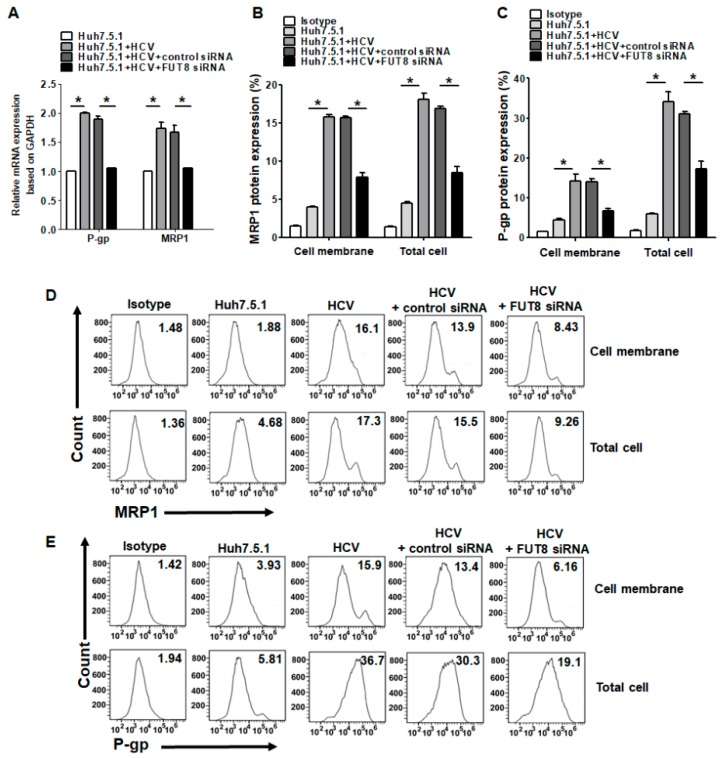
Effects of HCV infection on the expression of multidrug resistance-associated proteins. (**A**) Statistical analysis of qRT-PCR of the mRNA expression of MRP1 and P-gp after HCV infection in Huh7.5.1 cells with or without knockdown of FUT8. Statistical analysis of flow cytometry data for the expression of MRP1 (**B**) and P-gp (**C**) in Huh7.5.1 cells. (**D**,**E**) Representative data of flow cytometry analysis for the expression of MRP1 (**D**) and P-gp (**E**) in Huh7.5.1 cells after HCV infection or knockdown with FUT8. *p < 0.05.

**Figure 6:**
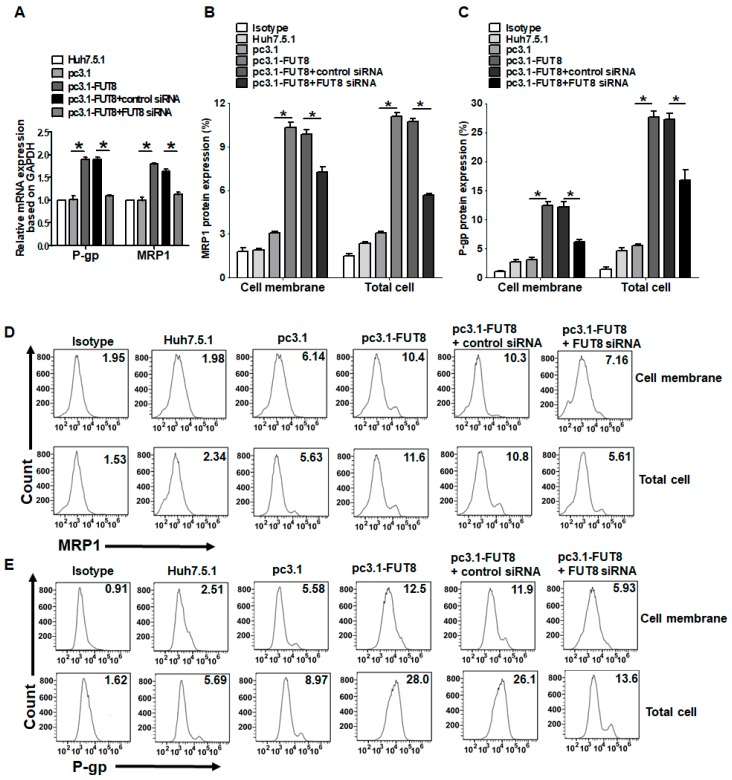
Effects of overexpression of FUT8 on the expression of multidrug resistance-associated proteins. (**A**) Statistical analysis of qRT-PCR of the mRNA expression of MRP1 and P-gp after FUT8 transfection in Huh7.5.1 cells with or without knockdown of FUT8. Statistical analysis of flow cytometry data for the expression of MRP1 (**B**) and P-gp (**C**) after FUT8 transfection in Huh7.5.1 cells. (**D**,**E**) Representative data of flow cytometry analysis for the expression of MRP1 (**D**) and P-gp (**E**) in Huh7.5.1 cells after stimulation with HCV infection. *p < 0.05.

To explore the mechanism of drug resistance mediated by FUT8, we detected the expression of the drug resistance genes in Huh7.5.1 cells. Overexpression of MRP1 and P-gp is often associated with substantially higher resistance to chemotherapy. Thus, we quantified the expression of P-gp and MRP1 in Huh7.5.1 cells and HCVcc-infected Huh7.5.1 cells. As observed, expression of P-gp and MRP1 mRNAs was significantly upregulated in HCVcc-infected Huh7.5.1 cells compared with Huh7.5.1 cells (*, *p* < 0.05; Figure 5A) but could be inhibited by knockdown of FUT8. The protein expression levels of MRP1 (Figure 5B,D) and P-gp (Figure 5C,E) in Huh7.5.1 cells both in the cell membrane and whole cell were determined by FCM and were consistent with the mRNA expression levels. We achieved similar results as those with HCV infection when FUT8 was overexpressed in Huh7.5.1 cells. Upregulated expression of the mRNA levels (Figure 6A) and protein levels of MRP1 (Figure 6B,D) and P-gp (Figure 6C,E) could also be seen when FUT8 was overexpressed in Huh7.5.1cells. These data indicate that FUT8 is an upstream regulator of MRP1 and P-gp. These effects caused by the overexpression of FUT8 could be reversed by the knockdown of FUT8 (Figure 6A–E).

### 3.5. Inhibition of P-gp and MRP1 Increases the 5-FU Drug Sensitivity after HCV Infection

**Figure 7:**
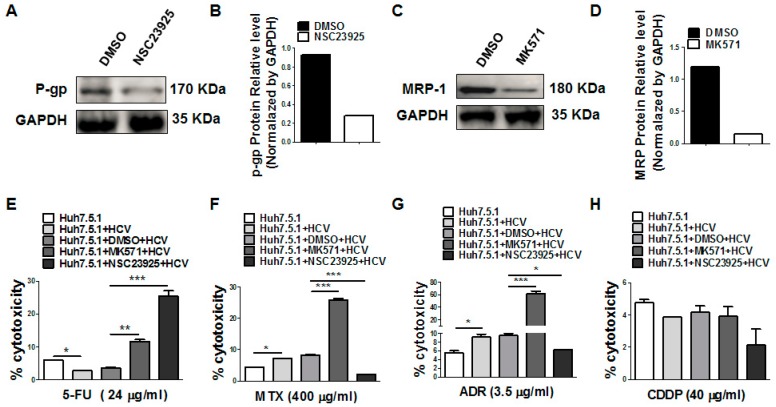
Inhibition of P-gp and MRP1 increased the 5-FU drug sensitivity after HCV infection. (**A**,**C**) Western blot analysis of P-gp and MRP1 expression in Huh7.5.1 cells in the presence or absence of NSC23925 (P-gp inhibitor) and MK571 (MRP1 inhibitor). Western blot quantification analysis is also listed as (**B**,**D**). (**E**–**H**) Analysis of the cytotoxicity of drugs (5-FU/MTX/ADR/CDDP) in HCVcc-infected Huh7.5.1 cells in the presence of P-gp and MRP1 inhibitors.

Next, we used the MRP1 andP-gp-specific inhibitors to detect the effects of MRP1 and P-gp on the different anti-tumor drugs in HCVcc-infected Huh7.5.1 cells. Western blot analysis confirmed that NSC23925 (P-gp specific inhibitor) and MK571 (MRP1 specific inhibitor) significantly decreased the expression of P-gp (Figure 7A,B) and MRP1 (Figure 7C,D), respectively. As expected, treatments with P-gp inhibitor NSC23925 and MRP1 inhibitor MK571 increased the 5-FU cytotoxicity and sensitivity to HCVcc-infected Huh7.5.1 cells (Figure 7E). MK571 treatment also increased, but NSC23925 decreased, the MTX and ADR cytotoxicity and sensitivity to HCVcc-infected Huh7.5.1 cells (Figure 7F,G). However, no effect—or a limited effect—on the cytotoxicity of CDDP was observed by the inhibition of P-gp and MRP1 (Figure 7H). These results indicate MRP1 and P-gp have different effects on these drugs (5-FU, MTX, CDDP and ADR). Only 5-FU drug sensitivity was increased by the treatment of both P-gp and MRP1 inhibitors.

### 3.6. NF-κB is Critical for FUT8-Mediated 5-FU Resistance in Huh7.5.1 Cells

**Figure 8:**
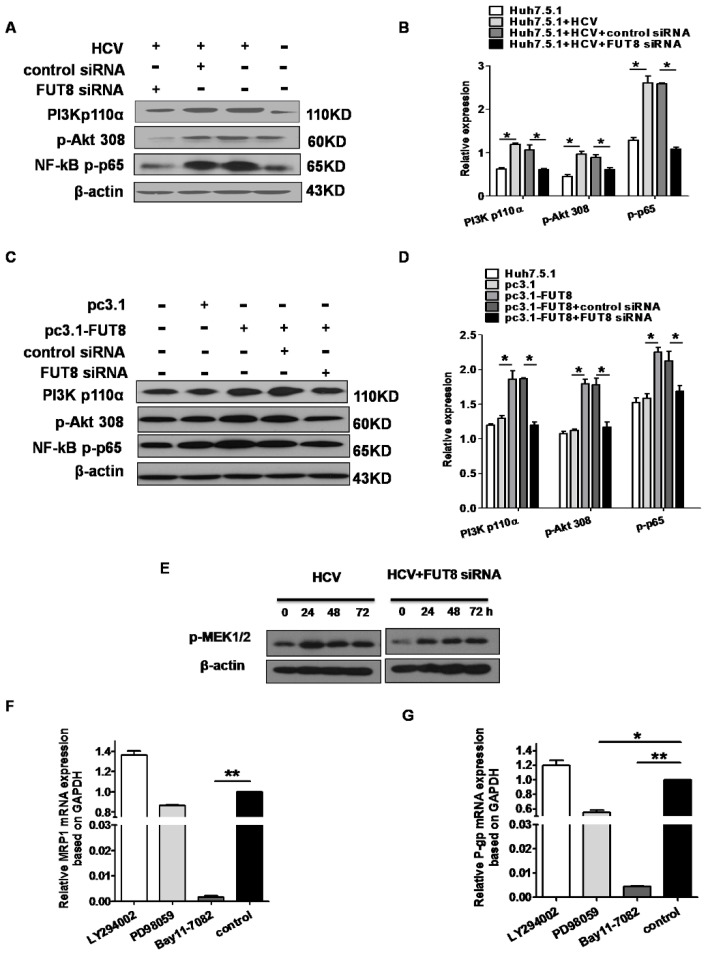
Analysis of the PI3K/Akt/NF-κB and MEK1/2 signaling pathways after FUT8 over-expression in Huh7.5.1 cells. Western blot analysis of the expression of PI3K/Akt/NF-κB signaling molecules after HCV infection (**A**) and FUT8 overexpression (**C**) in Huh7.5.1 cells. A representative blot from three independent experiments is shown here. The statistical analyses of (**A**,**C**) are also listed in (**B**,**D**). Different time point expressions of p-MEK1/2 (**E**) signaling molecules were detected by Western blot. QRT-PCR analysis of the mRNA expression of MRP1 (**F**) and P-gp (**G**) by the inhibitors of PI3K (LY294002), p-MEK (PD98095) and NF-κB (Bay11-7082) after HCV infection in Huh7.5.1 cells. * *p* < 0.05, control group vs. PD98059-treated group; ** *p* < 0.01, control group vs. Bay11-7082-treated group.

Since FUT8 is highly related to the drug resistance of Huh7.5.1 cells, we sought to determine the molecular mechanism involved in the function of FUT8. Given the critical role of the PI3K/Akt/NF-κB pathway in controlling cell drug resistance, we investigated whether the FUT8 gene activated the PI3K/Akt/NF-κB signaling pathways and whether PI3K/Akt/NF-κB had critical roles in HCV infection and FUT8-mediated drug resistance. Western blot analysis showed that the levels of PI3 kinase p110 (the catalytic subunit of PI3K), phosphorylated Akt (pAKT), and NF-κB p-P65 were increased by infection with HCV, which was then reversed by treatment with FUT8 siRNA (Figure 8A,B). We also found that the levels of PI3 kinase p110, pAkt and NF-κB p-P65 were increased after FUT8 transfection (Figure 8C,D).

We next sought to determine which molecules are critical for the induction of MRP1 and P-gp. As shown in Figure 8F,G, the expression of MRP1 and P-gp stimulated by HCV was significantly decreased by blocking NF-κB (Bay11-7082-treated group vs control, ** *p* < 0.01), but not by the inhibition of PI3K (no significant difference), which demonstrated that NF-κB was critical for FUT8-mediated 5-FU resistance in Huh7.5.1 cells, while PI3K is not involved. We found that HCV infection increased p-MEK expression and MEK inhibitor could significantly decrease the expression of P-gp, but not MRP1, in Huh7.5.1 cells (Figure 8E–G). These results indicated that FUT8-modulated multidrug-resistant (MDR) in Huh7.5.1 cells was, at least in part, MEK/NF-κB dependent, and NF-κB was critical for FUT8-mediated 5-FU resistance.

### 3.7. FUT8 Promotes the Proliferation of HCVcc-Infected Huh7.5.1 Cells through Activating the PI3K Signaling Pathway

**Figure 9:**
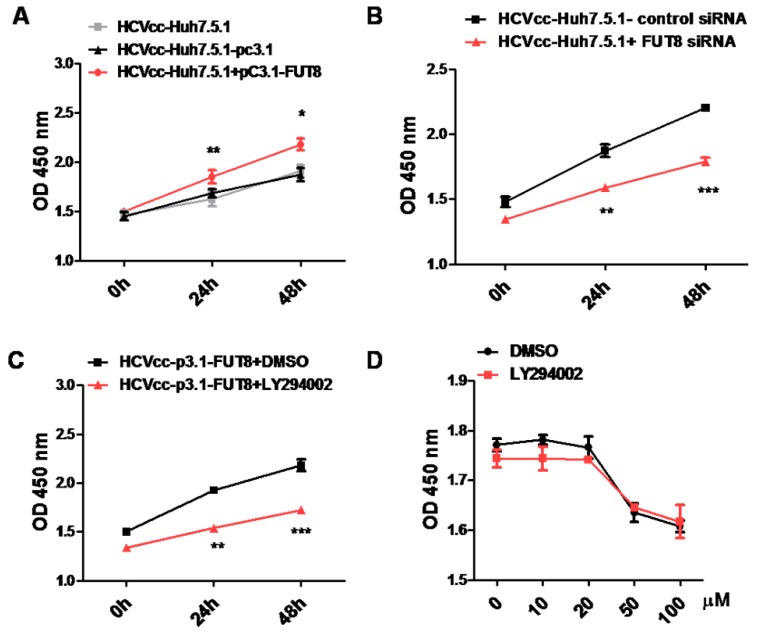
FUT8 promotes HCV-infected cell proliferation through PI3K. The effects of the overexpression (**A**) or silencing (**B**) of FUT8 on the proliferation of HCV-infected Huh7.5.1 cells were detected by CCK8 assay at different time points. (**C**) The effect of PI3K inhibitor LY294002 on the proliferation of HCV-infected Huh7.5.1 cells was detected by CCK8 assay at different time points. (**D**) Huh7.5.1 cells were pretreated with different concentrations of LY294002 or DMSO control reagent for 2 h, then washed and detected after 48 h incubation by CCK8 assay.

We further verified the effect of FUT8 on the cell proliferation and viability of HCV-infected Huh7.5.1 cells using CCK8 assay. As shown in Figure 9A,B, cell proliferation and viability was significantly increased by the overexpression of FUT8 (Figure 9A), and dramatically decreased by silencing of FUT8 (Figure 9B) compared to the empty vector treated group and the group with only HCVcc-infected Huh7.5.1 cells. In order to examine whether the PI3K signaling pathway is important in FUT8-mediated Huh7.5.1 cell proliferation, HCVcc-infected Huh7.5.1 cells were treated with PI3K inhibitor LY294002. Co-treatment with LY294002 (20 μM) markedly decreased FUT8 promoted-Huh7.5.1 cell proliferation compared with the group treated with DMSO alone (Figure 9C). Low/no cytotoxicity was observed in the presence of LY294002 alone at a concentration of 0-20 μM (Figure 9D). These results suggest that PI3K is involved in the FUT8 mediated the proliferation of HCVcc-infected Huh7.5.1 cells.

### 3.8. FUT8 is Highly Related to 5-FU Resistance in the Lewis Cell Model

**Figure 10:**
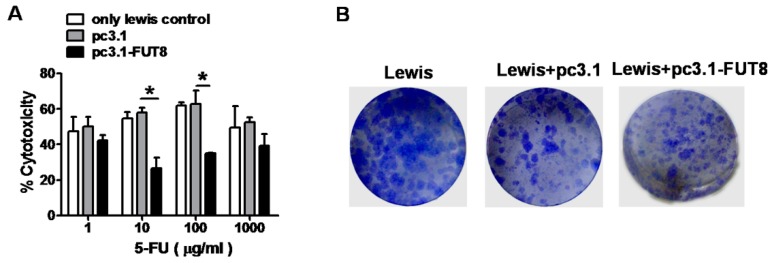
Overexpression of FUT8 enhanced the chemo-resistance of Lewis cells. (**A**) LDH assay of the effect of overexpression of FUT8 on the chemo-resistance to 5-FU in Lewis cells. * *p* < 0.05, pc3.1-FUT8-treated group *vs* lewis/pc3.1 groups. (**B**) The colony formation assay of the effect of overexpression of FUT8 in Lewis cells.

To confirm the function of FUT8 on the 5-FU resistance of tumor cells, we performed the same experiments using another tumor cell model: the Lewis lung cancer cell. The cytotoxicity of 5-FU decreased after overexpression of FUT8 in Lewis cells (pc3.1-FUT8-treated group *vs*. pc3.1-treated group, *, *p* < 0.05; Figure 10A). The clone formation test showed that many more colonies formed after transfection with FUT8 (Figure 10B). We can observe that the results from the Huh7.5.1 and Lewis cells were similar, and thus, we can conclude that overexpression of FUT8 is highly related to drug resistance in Lewis tumor cells.

## 4. Discussion

FUT8 transfers the fucose moiety from guanosine diphosphate-β-L-fucose to the innermost GlcNAc residue in N-glycans, denoted as core fucose [22]. Extensive literature indicates that alteration of glycosylation occurs during the progression of HCC [23,24]. Thus, FUT8 might have an important role in the development of HCC or in HCV infection. In previous research from our laboratory, we found that FUT8 expression was upregulated by HCV infection in Huh7.5.1 cells. It is very meaningful to investigate the mechanism of FUT8-mediated drug resistance in HCC, especially by HCV infection.

Firstly, our data showed that FUT8 enhanced the proliferation of Huh7.5.1 cells through the PI3K-NF-κB pathway (Figure 9). Similarly, other reports also showed that the FUT8 and FUT family were associated with the proliferation of tumor cells [9,25]. FUT8 promotes breast cancer cell invasiveness by remodeling TGF-β receptor core fucosylation [25] and drives the proliferation and invasion of trophoblastic cells via the Insulin-like growth factor 1 (IGF-1)/IGF-1R signaling pathway [26]. Our present work depicts a link between HCV-induced FUT8 and the cell proliferation of human Huh7.5.1 cells.

Four anti-tumor drugs (5-FU, MTX, ADR and CDDP) have been tested in our study. As a pyrimidine analog, 5-FU exerts its anticancer effects through the inhibition of thymidylate synthase (TS) and the incorporation of its metabolites into RNA and DNA [27,28]. MTX is a potent inhibitor of dihydrofolate reductase (DHFR), a key enzyme for intracellular folate metabolism, and functions to regenerate tetrahydrofolate from dihydrofolate [28]. CDDP is one of the most potent antitumor agents known, displaying clinical activity against a wide variety of solid tumors. Its cytotoxic mode of action is mediated by its interaction with DNA to form DNA adducts, primarily intrastrand crosslink adducts [29,30]. ADR is an inhibitor used for interrupting the transcription process and blocks RNA synthesis [31].

HCC easily acquires drug chemotherapy resistance. Many factors have been identified in the development of resistance to chemotherapeutic agents, such as elevated expression of drug efflux transporters; changes in drug kinetics; amplification of drug targets or the transition from epithelial to mesenchymal-like cells, which comprises genetic variation; and the tumor microenvironment [32,33,34]. Virus infection is also associated with tumor chemotherapy resistance [30,31,35,36]. However, few studies have reported on virus-induced abnormal glycosylation-associated tumor chemotherapy resistance. In the present study, we described for the first time that HCV-induced abnormal FUT8 leads to 5-FU drug resistance in human hepatoma Huh7.5.1 cells.

P-gp is responsible for decreased drug accumulation in MDR cells and often mediates the development of resistance to anticancer drugs. MRP1 is closely involved in MDR in several types of cancer, by transporting anticancer drugs across cellular membranes and reducing drug accumulation in the cells [37,38]. Our present results clearly showed that HCV-induced FUT8 increased the expression of P-gp and MRP1 (Figure 6), which consequently affected the 5-FU resistance of Huh7.5.1 cells (Figure 4 and Figure 7). Zhao Y. and Fujiwara et al. also proposed that the expression of P-gp and MRP1 in tumor cells was associated with resistance to 5-FU [39,40,41]. Our data showed that FUT8 knockdown, P-gp and MRP1 inhibitors increased the 5-FU drug sensitivity after HCV infection, which suggests that the resistance of 5-FU is associated with HCV-induced FUT8, MRP1 and P-gp.

Interestingly, our present data revealed that only 5-FU drug resistance is specific in HCV-infected Huh7.5.1 cells. We also found that FUT8 induced the 5-FU drug resistance of Lewis cancer cells. Whether other viruses, such as HBV, can induce 5-FU resistance needs further verification. We did not observe that HCV infection induced MTX, ADR and CDDP resistance. Different effects and possible different mechanisms of these drugs in HCV-infected Huh7.5.1 cells may need to be further investigated in future work.

We demonstrated that FUT8 induced anti-tumor drug 5-FU resistance mainly via the NF-κB pathway. NF-κB inhibitor dramatically suppressed the expression of both MRP1 and P-gp in the HCV-infected Huh7.5.1 cells (Figure 8F,G). Similarly, a study by Liu et al. also showed that the critical role of the NF-κB pathway involved in HBx protein induced 5-FU resistance process in HCC [42]. Based on our present study, we propose that the FUT8-NF-κB-MRP1/P-gp signal axis is involved in 5-FU drug resistance of Huh7.5.1 cells during HCV infection.

Since the function of FUT8 has been clearly elucidated in vitro, we will further verify the role of FUT8 in HCV infection and HCC using HCV-infected mouse models and clinical samples in future work.

**Figure 11:**
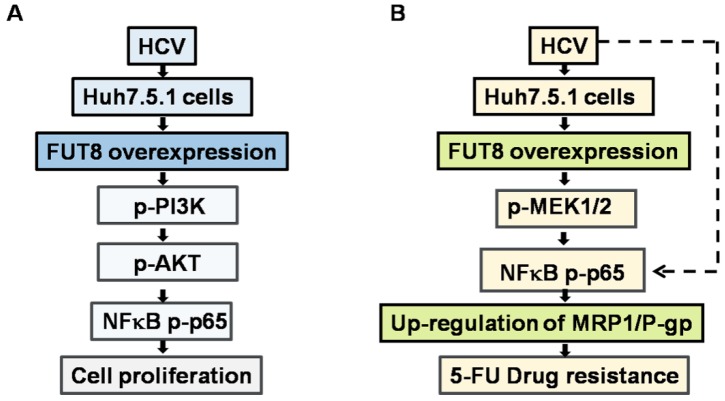
Putative model of FUT8 induced proliferation (**A**) and 5-FU drug resistance (**B**) in HCV infected human Huh7.5.1cells.

In summary, three major observations were made in this study (Figure 11). First, our data suggest that HCV-induced FUT8 promotes Huh7.5.1 proliferation by activating PI3K-AKT-NF-κB signaling. Second, HCV infection and FUT8 overexpression lead to 5-FU resistance of Huh7.5.1 cells. Third, NF-κB-MRP1/P-gp signaling is involved in the FUT8-induced 5-FU drug resistance of Huh7.5.1 cells during HCV infection. Our results may have far-reaching implications for the understanding of the mechanism of HCV infection and HCC 5-FU resistance from glycol-immunological aspects. FUT8 may serve as a therapeutic target to reverse chemotherapy resistance in tumor cells.
